# Soliciting stakeholders’ views on the organization of child and adolescent mental health services: a system in trouble

**DOI:** 10.1186/1753-2000-7-42

**Published:** 2013-12-23

**Authors:** Philippe Vandenbroeck, Rachel Dechenne, Kim Becher, Maria Eyssen, Koen Van den Heede

**Affiliations:** 1 Belgian Health Care Knowledge Centre (KCE), 55 Boulevard du Jardin Botanique, Brussels 1000, Belgium; 2 shiftN, De Hoorn Creative Minds, Sluisstraat 79/03/01, Leuven 3000, Belgium

**Keywords:** Adolescent, Child, Health services research, Mental health services, Organizational policy

## Abstract

**Background:**

Despite a high prevalence of mental health problems among children and adolescents Belgium, like many other Western countries, does not have a clear strategy for the organization of child and adolescent mental healthcare services (CAMHS).

**Methods:**

This paper describes stakeholders’ views on the organization of CAMHS based on a qualitative study. Ten in-depth interviews with high profile stakeholders were complemented by roundtable discussions (n = 30).

**Results:**

This diagnostic analysis illustrated that the system is in serious trouble characterized by fragmentation and compartmentalization.

**Conclusion:**

The findings create a sense of urgency that should be used to initiate a system reform of the Belgian CAMHS system.

## Background

In 2005, the World Health Organization (WHO) called for a national address of child and adolescent mental health concerns [1,2]. Reforms in most Western countries in the past have focused on the adult mental health sector. This sector, previously characterized by large isolated institutions, is gradually being transformed into a ‘balanced care model’. This implies that care is offered and delivered as close as possible to the patient’s living environment, and only if necessary in an institution [3].

Nevertheless, the child and adolescent mental health services (CAMHS)-sector requires a dedicated approach [2]. Firstly, the prevalence of mental health problems in children and adolescents is about 20%, and approximately 5% are believed to require clinical intervention [4,5]. Secondly, there appears to be a high degree of continuity between child and adolescent disorders and those in adulthood [5]. It is argued that appropriate interventions in childhood and adolescence can greatly enhance population health while improving outcomes for the young people involved [2]. Thirdly, it is widely accepted that an appropriate mental health policy for children and adolescents should specifically include a developmental perspective [6,7]. Finally, the CAMHS-sector developed much later than that for adults and does not have the same tradition of large isolated long-term inpatient service institutions [8].

Like in many other Western countries [5] also in Belgium there is no articulated child and adolescent mental health strategy. Therefore, the Belgian Ministry of Public Health commissioned the Belgian Healthcare Knowledge Centre (KCE) to perform a study that would offer input for a reform of the CAMHS-system. In this paper we report about a qualitative study that was part of this larger study [9]. The objective of this part of the study was to solicit the input from a range of stakeholders to understand what could be improved in the current CAMHS system. A broad approach was used, to acknowledge that supporting children and adolescents with mental health problems is not the responsibility of specialist mental health services alone. The scope of the research also included mental health services delivered at the primary care level by health care providers not specialized in child and adolescent mental health care, as well as services provided by neighbouring sectors such as education, child welfare and youth social care, services for disabled children and juvenile justice.

## Methods

The stakeholder engagement was organized around two participation events: in-depth interviews (July – September 2011) and exploratory round tables (September 2011). The roundtables aimed to confirm and complement the conclusions from the individual interviews. Even with the support of translation services it is hard to implicate different language groups (French/Dutch) at the same time in a technical, very interactive process. Therefore two roundtable discussions were held.

### Identification and selection of stakeholders

A purposive sample of stakeholders focusing on professional, expert and institutional stakeholders was composed. Children and adolescent patients were not directly implicated in the process. The user perspective was included in the process by way of representatives of parents and/or patient organizations and self help groups. Desk research and punctual information collected from key informants from our existing network were used to compile a long list of relevant CAMHS stakeholders. The 1st draft of the long list was screened using geographic as well as profile criteria (i.e. *practitioners* including private and public sector, child psychiatrists, child psychologists, youth justice professionals, pediatricians, general practitioners and school professionals; non-governmental organizations (*NGO’s*) including patients/children, and family rights advocate; *administrators and policy makers*: managerial functions in public/private institutions including government agencies, umbrella organizations representing the management and administration of CAMHS).

From this list, people were invited to the roundtables and in-depth interviews. *The interviews* focused on a small sample of 10 stakeholders which were known to be opinion leaders and to have considerable political influence in the CAMHS field.

The selection for the *round tables* aimed to have 15–20 participants in each language group with a balanced mix of profiles. If people were unable or declined to participate, people with a similar profile were invited.

### Process

*The interviews* were semi-structured (based on an interview guide, including open-ended questions). They explored interlocutors’ views on the current problems and bottlenecks in the CAMHS system. All conversations were face-to-face, recorded and fully transcribed. The interview round started in July and finished early September 2011 (before the roundtables were held).

*The roundtables* (1.5 h each) were organized around the question: “What image or metaphor captures, from your point of view, the core of the existing CAMHS system in Belgium?” Metaphors give an insight into the stakeholders’ unique perception of their situation and their goals [10]. As such, this question focused on eliciting participants’ views on the strengths and weaknesses of the present system.

Although the main aim of the interviews was to identify the friction points in the existing CAMHS system also some solution elements emerged. During the interviews this was done implicit while during the roundtables a specific question was asked. Participating stakeholders were then invited to propose their top 3 of interventions to improve the existing CAMHS system. They were invited to go beyond a proposed list of 10 if they wanted to. The proposed list of interventions, based on ‘Systems of Care [11]’ included:

1. Developing comprehensive home-and community based services and supports;

2. Developing family partnerships and family support;

3. Providing culturally competent care and reducing unmet need and disparities in access to services;

4. Individualising care;

5. Implementing evidence-based practices;

6. Coordinating services, responsibility and funding to reduce fragmentation;

7. Increasing prevention, early identification, and early intervention;

8. Strengthening early childhood intervention;

9. Expanding mental health services in schools and other adjacent sectors;

10. Strengthening accountability and quality improvement or to suggest other priorities.

### Data analysis

To structure the ‘diagnostic’ output of the interviews the ‘systems ladder’ of Meadows was used as guiding framework (See Table 1) [12]. It includes a hierarchy of 12 so-called ‘places to intervene in a system’. Blockages or malfunctions at these points can block the system’s functioning while interventions at leverage points can have a significant impact on the system’s behavior. The systemic nature of the framework lies in its proposed hierarchy of levels that have more or less structural impact on the system. For example, changing the goal of a system (Meadows ladder 3) will have a profound influence on its functioning, whilst merely trying to improve some parameters (Meadows ladder 12) is, from a systemic point of view, a more superficial intervention. In this study the 12 categories will be used as a framework to structure information from a diagnostic point of view, i.e. to map out what is wrong with a system. For each of the categories of the Meadows ladder we summarize the results including a statement and corresponding “interview quotes”. In addition, we describe also the main categories of ‘solution elements’ that emerged from the interviews with supporting “interview quotes”. The interview quotes have been translated, shortened and are sometimes paraphrased. The full quotes in the original language are available online both in the original language (Dutch or French) as well as English [13].

**Table 1:** **Leverage points ‘Meadows ladder’ or places to intervene in a system**^**1**^

**Label**	**Explanation meadows**	**Systemic problems in Belgian CAMHS**
**12) Numbers**	Constants and parameters such as subsidies or taxes.	• Lack of financial resources
		• Inappropriate allocation of resources to CAMHS relative to the investment in mental health services for adults
**11) Buffers**	Sizes of stabilizing stocks relative to their flows. These are aggregates of various types (people, finances, materials) that determine the system’s behavior. Buffers that are small relative to their flows may lead to system instability. Large buffers may compromise the adaptiveness of a system.	• Lack of a workforce (provider network) that is prepared to provide state-of-the-art services and supports
**10) Stock-and-flow structures**	Physical systems and their nodes of intersection. This concerns the capacity of infrastructural elements that sustain a flux or flow in a system.	• Lack of service capacity
		• Limited range of service
		• Lack of home and community-based services
		• Overreliance on inpatient services
**9) Delays**	Lengths of time relative to the rates of system changes. Delays in feedback processes can significantly determine the behavior of a system, often leading to instability (oscillations) if they are out of synchronisation with the speed with which the system changes.	• Lack of capacity and saturation of services and resultant significant waiting lists for care
**8) Balancing feedback loops**	Strength of dampening feedback loops relative to the impacts they try to correct. These are dynamic forces that keep the system near equilibrium, in much the same way as a thermostat keeps a room’s temperature near a desired temperature.	• Reliance on inappropriately services due to lack of service capacity
		• Pockets of excellence in service delivery approaches that are not adopted and implemented system wide
		• Isolated services created to reduce pressure on the CAMHS system that result in additional fragmentation
		• System inertia and resistance to system reform
**7) Reinforcing feedback loops**	Strength of reinforcing, driving loops. These are dynamic forces that move the system away from an equilbrium (leading typically to phenomena of exponential growth).	• Continued growth in children and families’ demand for mental health services
		• Lack of coordination within and between sectors and both the system and service delivery levels exacerbate capacity problems and compromises clinical and functional outcomes for young persons and their families
		• Waiting lists lead to users’ demand-inflating strategies to access the system
		• Reinforcing demand-driven dynamic of increasing specialization and fragmentation in care services for young people.
		• Lack of strategies to address cultural and linguistic differences and disparities in access to and the quality of services
**6) Information flows**	Structure of who does and does not have access to information. Information flows are fairly obvious and easy to understand (whilst not necessarily easy to remedy) determinants of a system’s performance and behavior.	• Fragmentation at the system and service delivery levels
		• Lack of structured and coordination flows of information
**5) Rules**	Incentives, punishments, constraints, typically embodied by regulations of all sorts.	• No clear focal point of responsibility, management, and accountability at all levels
		• Systemic focus on “beds” and hospital-based services rather than a full range of services and supports
		• Lack of data for data-based decision making and continuous quality improvement at both the system and service delivery levels
**4) Self-organization**	Power to add, change or evolve system structure. This essentially concerns system features that allow it to learn and to adjust its structure and functioning to outside disturbances.	• Fragmentation of services both within the mental health sector and across other child-serving sectors
		• Focus on the child in isolation rather than in the context of the family and the wider environmental context
		• Lack of training of mental health professionals on a family focused and “ecological” approach to service delivery
**3) Goals**	Purpose or function of the system. This refers to the explicit or implicit goal(s) espoused by the actors working in and governing the system.	• No clear, agreed-upon goals for the CAMHS system
		• Lack of clear, agreed-upon desired outcomes for the CAMHS system
		• Lack of an appropriate focus on young persons across the developmental spectrum
		• Lack of a balance between treatment for young persons with identified mental health conditions and a “public health approach that also includes mental health promotion, prevention, and early identification and intervention
		• Lack of specification of a value-based practice approach for the entire CAMHS system
**2) Paradigms**	Mindset out of which the system arises (its goals, structure, rules, delays, parameters). This refers to the basic norms and values which give meaning to the system’s goals and functioning.	• Lack of family and youth partnerships at the system and service delivery levels
		• Lack of family-driven, youth-guided care
**1) Transcending paradigms**	Which is the ability to move flexibly between paradigms^2^.	Not applicable

^1^The 12 ‘places to intervene in a system’ are usually presented in an inversely numbered way (ordered from the less systemic to the more systemic).

^2^We have chosen not to include this level in the analysis as it goes beyond the scope of this study.

The results of the roundtables were summarized; an inventory of all participants’ responses can be found elsewhere [13]. The outcomes of the roundtables were used to confirm and complement/the conclusions of the interviews in a larger sample of stakeholders involved in the CAMHS system.

## Results

### Sample description

Ten stakeholders were interviewed in-depth (5 French-speaking and 5 Dutch-speaking): 6 child psychiatrists, 2 psychologists, 1 representative of a patients/children, and family rights advocate organization and 1 policy maker.

Thirty stakeholders participated on *the roundtable discussion* (13 Dutch speaking; 17 French speaking). There were 16 health care professionals representing different settings such as outpatient care, inpatient care, academic settings, etc. The majority were child psychiatrists (n = 10). Other profiles represented were psychologists (n = 2); general practitioners (n = 2); pediatricians (n = 2). Additionally, there was 1 representative of each of the following settings: juvenile justice, education, disability care. There were also 2 representatives of patients/children, and family rights advocate organizations and 9 stakeholders representing policy makers and administration.

### Diagnostic output from the interviews

#### Numbers

Stakeholders refer to the significant discrepancy between the prevalence of mental health problems in this age bracket and the share of the total mental health budget it is entitled to. It was estimated that less than 5% of the total mental health budget is allocated to CAMHS.

**Compared to the adult sector, the CAMHS is significantly underfunded:** “I once calculated that only three percent of the national budget for mental health care was allocated to youth. While we are dealing with 20 to 25% of the total population”.

#### Buffers

A key buffer is the shortage of child psychiatrists. The profession is deemed unattractive for financial reasons and because the work is hard and stressful. Many psychiatrists choose to work in a private practice, focusing on less complicated cases (‘cherry picking’). Conversely, there is an oversupply of clinical psychologists. However, in Belgium they are not recognized as ‘health workers’ and hence not reimbursed by social security. The supply of other, perhaps less traditional types of CAMHS providers has not been well developed.

**Mental illness continues to be stigmatised in our society. This has implications for the attractiveness of the profession:** “Twenty years ago, we used to have much more candidates. Today there are not enough candidates to fill the training positions. I really think there is still a taboo around mental illness in our society. It is not seen as a genuine illness. People do not have the same respect for it as for a cardio-vascular disease”.

**Child psychiatrists choose not to work in hospitals but to establish their own practice that focuses on not too difficult cases as it is easier and more lucrative:** “Child psychiatrists don’t want to work in a hospital anymore. It is much more financially attractive to have an independent practice and to work with children that do not have too complex problems”.

**The official reimbursement of clinical psychologists remains a controversial point:** “There is a lack of child psychiatrists and oversupply of clinical psychologists. The fee-for-services system transfers final responsibility and financing to child psychiatrists only, with psychologists in a supporting role and being paid via the psychiatrists. This is a very hierarchic way of working that is not in accordance with the way it should actually work”.

#### Stock-and-flow structures

There are long **waiting lists** both in outpatient and residential CAMHS services. The saturation of the CAMHS system is for many stakeholders **the key factor that compromises the effectiveness of the system** and that contributes to its negative image. Overall, traditional outpatient, inpatient, and residential services are the center of gravity of the CAMHS system. Flexible home-based and community-based mental health services and supports that are able to provide alternatives to treatment in inpatient and residential settings have not been widely put in place. Also the lack of emergency and crisis facilities is acute. These capacity problems bounce off one another and reinforce each other.

**The CAMHS system is unable to cope with demand. There are long waiting lists everywhere in the system:** “I believe that the waiting lists have been the biggest problem over the last few years. Certainly in the residential facilities and in daycare centres there are few opportunities for children and adolescents to be admitted in a crisis situation. So demand increases but neither youth care nor mental health facilities have developed an appropriate response. We are not structurally organized for these crisis admissions. I think this has been the most striking observation during my whole career, which goes back for almost 30 years”.

**Ambulatory services are underpowered:** “What strikes me as the most acute need in the CAMHS system is the absolute shortage of outpatient services. That’s quite obvious from the long waiting lists we are coping with”.

#### Delays

The delays in the CAMHS system are a result of saturation of the care system, with ubiquitous waiting lists as a result (see: stock-and-flow structures, balancing feedback loops, reinforcing feedback loops).

#### Balancing feedback loops

The CAMHS system is under pressure. One reaction on the capacity problems is **‘passing the buck’**, whereby saturated services pass on youngsters to other, more or less adequate, services. Another reaction is to implement localized initiatives to take pressure off of the system. Whilst these do help in meeting certain needs and offer opportunities for service innovation, stakeholders point out that typically these new capacities also are quickly saturated. The proliferation of **these isolated initiatives,** however well intended and executed**, contributes to the fragmentation of services** and of available financial resources. Furthermore, based on the experience of these new services quickly reaching capacity, actors in the sector are reluctant to undertake further initiatives. Thus, there are balancing loops operating at two levels. At a sectoral level, these isolated initiatives reduce the pressure on the overall system somewhat by acting as safety valves. However, they represent a temporary ‘fix’ and many contribute to the system’s inertia and resistance to reform. Furthermore, also the **absence of a strong voice of an empowered family network** can be considered as a balancing loop that reinforces the system’s inertia.

**Isolated initiatives take pressure of the system but are quickly saturated:** “If I would take on more emergencies our department would run at full capacity all the time. But as so often, once the extra capacity is there, in two months time it is saturated. Taking the responsibility entails the risk is that one attracts all the misery of the entire French region”.

**Fragmentation leads to facilities being stretched thinly and being under resourced:** “There are many laudable initiatives. But at a certain point all these compromises lead to a budget that is being very thinly stretched. One grants resources here and there but with a risk of fragmentation and throwing in disarray the provision of care”.

**There is no patient organisation that works for and with parents of children with mental health problems:** “There is no patient organisation for children and adolescents. We tried, about ten years ago, but parents don’t want to share their experiences openly with others”.

#### Reinforcing feedback loops

The CAMHS system is in many respects a system that is governed by reinforcing loops, steadily pushing the system away from a desirable, stable level of performance. The **capacity problems reinforce one another**. Stakeholders acknowledge that there is a dynamic of ‘passing the buck’ from one service to another: the lack of ambulatory capacity puts more stress on crisis facilities, which are quickly saturated and send children onwards to residential facilities where they don’t belong. This leads to inappropriate and inefficient use of the available capacity of expensive, residential facilities. In addition, the system’s ineffective response results in poor clinical and functional outcomes for both young persons and their families. These issues do not only manifest themselves within the sector of CAMHS but also in adjacent sectors such as youth care and juvenile justice.

**The lack of co-ordination between first line and deeper end services leads to escalation in children’s troubles and disturbances:** “The big frustration of General Practitioners is that when they send a youngster to a crisis facility, they get the message ‘there is no indication, he doesn’t fit in our group, etc. So they get them back and then later they are reluctant to send them onwards. As a result problems escalate to manifest crises and then you need the heavy residential facilities”.

Another important, exogenous reinforcing loop is an ever **increasing demand from young people and their families for mental health services**. This demand results from many coalescing forces operating at the level of broader society. These societal processes have not been fully elucidated in these interviews. However, stakeholders noted that the presence of mental health problems appears to be increasing and presenting problems are increasingly serious and complex.

**The number of young people that rely on the CAMHS system keeps on growing:** “It’s a fact that over the last couple of years there has been an increase in kids and families in suffering. That is an observation that applies across sectors: health care, youth care and juvenile justice. All these lines are saturated by the number of children faced with difficulties in their families”.

A demand-driving factor is the so-called **‘target group’ approach**, whereby services are targeted to particular diagnostic groups or to particular types of problems, such as youth who have committed offenses. This is a clinical approach which distinguishes a progressively finer catalogue of mental and behavioural problems. However, categorizing and labelling these mental health challenges creates and reinforces own demand both from users and providers. This approach tends to limit services to particular priority groups and constrains the availability of help to the entire group of young persons needing mental health care and their families. Furthermore, the institutional response leads to greater fragmentation in the service landscape.

**A clinically informed target group policy creates its own demand and sometimes assessments are tweaked in order to squeeze youngsters in a category where there is spare capacity:** “There are, for instance, separate care circuits for kids with delinquent behaviour, autism or ADHD. For all other kids there is no dedicated support. So if you want to get help, you have to behave like a criminal, or an autist or a person with ADHD. The result is that the number of ADHD and autism diagnoses is rapidly increasing”.

Lack of attention to cultural and linguistic differences among the communities in Belgium also leads to variable service delivery across the country and inappropriate services for each group. **Disparities in access to and in the quality of care** are experienced as a continuing problem for the CAMHS system.

**Lack of attention to cultural and linguistic differences among the communities in Belgium also leads to variable service delivery across the country and inappropriate services for each group:** “We are probably the only bi-lingual hospital in Brussels. French-speaking hospitals are having a hard time with Dutch-speaking patients. I really dream of small facilities (‘cells’), dispersed in the city and where people are always taken care of, adequate resources are present and both languages are spoken”.

By their very existence, **waiting lists spur demand**. People, being aware of the bottlenecks, often register at several entry points at once hoping to get quicker access quickly inflating waiting lists beyond realistic proportions. Some stakeholders think that a centralised registration system for children entering the care system might create much needed transparency and more organized pathways to care. Care needs to be taken that information management tools do not lead to stigmatization as an unintended consequence.

The existence of waiting lists leads people to demand-inflating strategies to access the system: “The waiting lists are relative. The debate is too linear, as if the numbers represent reality whilst everyone knows that people put their kids on the list in four institutions to play it safely. The absence of a central registration point implies that it is difficult to put in a place an effective policy to deal with that situation”.

#### Information flows

The fragmentation of the CAMHS system is reflected in **a lack of structured and co-ordinated information flows between the actors in the system**, and between the sector and adjacent areas of youth services. The compartmentalization also affects informal networks within and across sectors. An important missing element is a reliable assessment of what the regionally-based demand for CAMHS services is.

**Lack of a centralised registration system:** “If you want to respect the rights of children, you have to make sure that facilities can continue to pursue an ‘open door’ policy, that systems are not saturated by insistent searchers that are always trying to find a new access point. In Holland you can have a ticket for an ADHD-investigation. But if that has been done you can’t reapply for a period of three years”.

#### Rules

Stakeholders point out that the **hospital-centric CAMHS system** is governed by an elaborate regulatory framework that governs financing, the exercising of the medical and other mental health professions across disciplines, the management of a vast and costly infrastructure to support these services, and the rights and duties of patients and mental health and legal professionals respectively. This elaborate system of rules is not centrally administrated but rather is fragmented across different institutional levels (federal, regional and local) and sectors (mental health, youth care, education, juvenile justice). This leads to complexity, compartmentalization, and a desire of influential actors to maintain to the status quo. In particular, stakeholders singled out the basic datum that the majority of financial resources are allocated to beds (i.e. residential facilities managed by psychiatric hospitals). Maintaining the ‘bed’ as the pivotal element of a mental health care system significantly constrains the system’s ability to evolve towards a more integrated and effective approach to service delivery.

**Hospital-linked resources are not flexibly allocated because of norms and regulation:** “Nowadays hospitals keep thinking in terms of units, the staffing ratios and the money that is associated with that. They hesitate to allocate that budget outside of the hospital, also out of fear of being reprimanded by inspection authorities”.

Policy making is hampered by the **absence of a transparent evaluation framework**. Evaluation methods are either non-existent or inappropriate, adding to the administrative burden of practitioners and constraining the ability for data-informed decision making and continuous quality improvement at both the system and service delivery levels. Particularly the Minimal Psychiatric Data Set is singled out as missing the mark.

**There is a lack of appropriate evaluation methods:** “We have been doing the Minimal Psychiatric Data Set for 10 years and it has absolutely no added value. It takes me a quarter of my time as a psychiatrist to fill that into the computer which crashes 75% of the time because their software is not very stable. We’ve been entering these data and nobody has been able to tell us at the 10th anniversary of the system, what was being done with them. Nobody has published anything which could help us to focus our work”.

**There is no assessment of the overall effectiveness of the existing CAMHS system:** “There is very little research on the effects of CAMHS services offered. Children arrive at the cabinet of someone who calls himself a therapist. He or she does something with the child, or not. Does anything change? As far as we know the effectiveness of the system is zero. In the best of cases it’s as effective as the placebo effect”.

#### Self-organization

The capacity of a system to self-organize is its capacity to learn and to adjust its structure and operation in response to outside disturbances or internal stresses. One of the most conspicuous features of the Belgian CAMHS system as pointed out by the respondents is its level of **fragmentation and compartmentalization**. This makes it difficult for users and professionals to navigate the system, to exchange information and to develop a shared vision of purpose and governance of the system. The result is that the system generally lacks **the capability of adjusting to changing conditions**. The CAMHS system’s compartmentalization is to a significant extent determined by institutional factors and by legacy infrastructures and vested interests.

**The growing differentiation in mental and behavioral problems leads to institutional fragmentation which is harder to govern and co-ordinate:** “At the end of the day you are sitting with sixteen professionals around one child. And you have constantly groups that are making a case that something has to happen around a certain facet of the problem. That’s a problem of clustering. And the increasing regulation implies that people are keeping an eye on what they don’t have to do. One organisation says: we are focusing on very small children. Others specialise in teenagers. There are centers for drugs, for traumapathology. The field is further parcelled out. But who is steering this centrally? Who is evaluating all these partial contributions?”

#### Goals

The goal of a CAMHS system entails three key dimensions: scope, developmental perspective and target or population based approach. Is the **scope of the system** focused on the child only or on the child, family and relevant social environment? Several stakeholders said that the existing system is too centered on the child in isolation without consideration of the family and the environmental context in which the child functions (i.e., school and community).

**The system is too child-centered and does not focus enough on its social environment, particularly the family:** “Take a classic example. A child has a cognitive disharmony. Doesn’t feel well at school. There are learning difficulties. Small emotional problems develop into relational problems. Nowadays parents don’t know how to handle a normal child, much less a child with complications. And there psychiatrists need to accept to work a little more ‘*orthopedagogically’* with a family. Because one loses a lot of time with very child-focused treatments whilst disregarding the psycho-educational context with the family”.

The **developmental perspective** concerns the fact that the needs and challenges of young persons evolve as they move from early childhood to young adulthood. To what extent is a mental health care system willing and able to adapt interventions to different stages of the developmental spectrum (specifically to young children and their families and to youth in transition to adulthood)? Stakeholders saw too few services that take this temporal perspective into account.

**There is very little in terms of initiatives or infrastructures that take into account a developmental perspective:** “In my experience care models need to take into account age brackets of about 6 years: 0–6 covers the question of development, 3–9 is the question of learning, 6–12 is childhood and hence the issue of the relationship to the parents, 9–15 is puberty and the management of sexuality, of the paternal function, the process of positioning with respect to the law, of respecting the collective, to live together. Then there is 12–18 years old. Most adolescent services focus on this age bracket. I continue: 15–21 is the period of orientation, life choices, partner choice, etc. And 18–24 is the category of ‘young adults old adolescents’. These age brackets have to be served by specific projects. But I see very few of these specific projects”.

A third key choice revolves around the distinction between a **‘target group’ approach versus a ‘population approach’** that sees the improvement of the psychosocial skills of all children (those with and without mental health problems). They pointed out the need for a balance between serving young persons with diagnosable disorders and a broader ‘public health approach’ that also includes strategies for mental health promotion, prevention of disorders, and early identification and intervention in addition to treatment for young persons with identified mental health conditions and their families.

**At this stage much of CAMHS is driven by a clinically-oriented target group approach. A population-oriented model investing in the general wellbeing of all children is more appropriate. A clinical approach can be grafted onto this population approach but should not be leading:** “In the population paradigm, approaches are deployed to support the whole population. For children this boils down to air, nutrition and education. We can’t do very much about genetic predisposition, maybe for the better. We can do something about those contextual factors. Another thing is: make people stronger instead of more dependent. The clinical model makes people dependent. Don’t pollute schools with the clinical model. If kids are difficult to handle at school, make teachers stronger to deal with that situation. Don’t immediately think ADHD. If nothing works and it breaks down, than a clinical intervention maybe appropriate. Also I don’t believe in the effectiveness of screening. It is too aspecific and the risk for false positives or false negatives is too large”.

From a policy standpoint, there is no clear, agreed-upon goal for the CAMHS system. Without being anchored in a clear understanding of its goals, the system is driven by the interests of institutions instead of the needs of young persons and their families. Given the lack of clearly defined goals, there is also a lack of clearly defined desired outcomes for the CAMHS system to be used to design the system and to deliver the services and supports needed for achievement of the specified outcomes.

**There is no overarching, inclusive model of the CAMHS and youth care sectors to guide policy:** “Why is child-abuse relegated to youth care but when it leads to unpleasant consequences it becomes child psychiatry? At government level there is no inclusive model. This is an essential paradox: how can you expect to come to an integrated model when the management does not happen from an integrated model?”

#### Paradigms

The shape of the CAMHS system is a reflection of fundamental views held by the medical profession (and by extension by the entire society). There seems to be a consensus that **children cannot be considered as ‘little adults’.** The concept of mental health for this group needs to be refined and made explicit and taken as the basis for a care system. On the other hand medical professionals have a hard time considering children and adolescents as stakeholders regarding their own troubles, and hence as partners and co-creators of their own care trajectory. The existing CAMHS system is traversed by the idea of guilt (of parents, of society vis-à-vis children) and victimhood. It would be more appropriate to relinquish these notions in favour of a concept of responsabilization, in which a social collective takes charge of a process of resilience, healing, and improved functioning. Finally, the fundamental right of all children and families to effective services and supports and to drive their own care is seldom taken as a cornerstone of a health care system.

**Children are not ‘little adults’. They have specific developmental needs. The concept of children’s mental health needs to be clarified and taken as a cornerstone for a care system:** “Children are not little adults. There is the dimension of development. The way a child perceives the world is very different. Acting as if children are adults is doing them a disservice”.

**The rights of children should be a foundational element in determining the kind of care system that ought to be developed; children and their families ought to be in the driving seat, not the care infrastructure:** “The Convention on the Rights of the Child has to play an important role. This says that each child needs to be offered a comparable level of care, whatever the circumstances. That is not the case in our care system. That is a consequence of this target group approach. Respect for the Convention means that children are not prematurely put into target group but that they are guaranteed that their development will be put in a broad perspective”.

### Diagnostic output from the roundtable

In this paragraph we summarize the results per round table. An inventory of all participants’ responses can be found elsewhere [13]. The images evoked by the participants to the **Francophone roundtable** reveal the following strengths and weaknesses of the CAMHS system:

•*Strengths*: Complexity, diversity; Pockets of goodwill, creativity, and efficiency.

•*Weaknesses*: Lack of accountability, control, instability; Rivalry, lack of collegiality, coordination; Congestion and saturation leading to frustration, confusion, isolation and loss of meaning; Lack of political vision, short-termism, leading to stagnation; Lack of transparency hence difficult to navigate for users and professionals, no feedback; Lack of resources; Inability to adapt, dwindling degrees of freedom; Inability to cure, to fulfill its most basic purpose; Inability to resolve the tensions of a stressful, contemporary society; Source of stigmatization.

The images summoned by the participants to the **Dutch-speaking** roundtable point to the following strengths and weaknesses of the CAMHS system:

•*Strengths*: Diversity, goodwill, expertise; Potential for learning, potential for establishing new connections; Pockets of efficiency; A discernible desire for reform.

•*Weaknesses*: Overall ineffectiveness of the system; Unattractive, inhospitable and intimidating character; Subject to taboos and stigmatization; Difficult to access, to navigate, to get out of the system, lack of transparency for outsiders; Complexity, fragmentation, chaos; Lack of an overall vision, of appropriate controls to steer and assess the quality delivered by the system; Subject to rivalries and lack of co-operation.

### Solution elements emerging from in-depth interviews

The 10 interviews with stakeholders not only yielded rich insights into the current problems and bottlenecks in the CAMHS system but also allowed to explore interlocutors’ views on what potential solution elements could be. The solution elements drawn from the interviews were categorised in four broad areas:

•Category 1: Development of cross-sectoral care circuits.

Institutional fragmentation is at the root of the CAMHS system’s inability to address the pressure it is confronting. This awareness in the interview sample translates into a plea for a more sectorally and cross-sectorally integrated CAMHS system. This entails a move from hospital-centric to **regionally-managed care circuits** where, depending on locally defined needs, also youth care, schools, peer support and others. The **‘outreach’ experiments** that have been put in place in Belgium under the aegis of daycare centres since 2006 are considered to be valuable precursors.

"Demand-led and subsidiarity are key concepts. Subsidiarity means that **care is provided at the least intrusive level**. But that is only possible if you can manage the **whole trajectory**. When you do not have to say: I don’t have those facilities in my trajectory. Demand-led means that the needs are central, not the protocol. And it has to rely on genuine contact”.

"I think that the **hospital has a place, but in a network. Not in a structure that is made by itself**”.

"I think that the **outreach model**, which relies on a very intensive collaboration between a daycare centre and residential facility and the family situation or other services, is a very good model. I believe strongly in it, also because it appears to be able to avoid children ending up in residence”.

•Category 2: Broadening of the service array, notably development of home and community-based services;

Cross-sectorally integrated care networks have to be able to offer a comprehensive array of services so that they can function in a genuinely demand-led way. Interviewees also refer to this as the principle of ‘subsidiarity’, meaning that, whenever possible, ‘lower level’ (less complex) home-based or outpatient services are relied on instead of costly and scarce residential services.

"Ideally a trajectory is made, with emphasis on **outpatient services**. However, in reality we see that services are quite limited. There is nothing for chronic patients – a young person that has to stay in a residential facility from 6 to 18 years old. Outpatient is quite limited. Day care is limited as well and outreach projects have only 2 full time staff. So, if you want to organise a concept of tailor-made care, these are the building blocks. There are gaps and imbalances”.

•Category 3: Development of additional crisis and emergency capacity;

Lack of emergency and crisis capacity is acutely felt in the field and in response interviewees argued for the creation of supplementary, strong and multidisciplinary crisis facilities.

“Emergency situations need to be dealt with between youth care, emergency services of psychiatric hospitals and physical disability care. **Multidisciplinary teams have to be created with representatives of all these agencies**”.

•Category 4: Development of clear entry points to the system

Children and adolescent mental health professionals are concerned about the ill-structured access to the CAMHS system. This ought to be better structured by either streamlining the entry gates or by bolstering the mental health expertise at the various points of contact.

"Each age-based category ought to have a trajectory, with **dedicated entry gates**, first to the outpatient services, then to home-based, then day care and finally to residential services. The majority of youngsters ought to be serviced in outpatient care”.

### Solution elements emerging from roundtable discussion

In identifying appropriate interventions for improving the system an ambition to realize a sectorally (between outpatient and residential services) and cross-sectorally (between mental health and adjacent services) **more integrated care system** dominated (intervention number 6 from the proposed list of 10 interventions, see Methods).

A second key intervention was to make the system more **child and family-centered** by providing customized (personalised) care, preferably in home and community settings, and by establishing family-partnerships (interventions numbers 1, 2 and 4).

A third point of gravity was the **strengthening of prevention, detection and early interventions** (intervention numbers 7, 8, 9). However, stakeholders in both language groups were acutely aware of the potential unintended consequences (lock in, stigmatization) of early detection.

Some of the respondents advocated **evidence-based practices** (intervention nr 5 and also 10) strongly but stressed the importance that it should understood as not to exclude therapeutic approaches that yield promising results but have not been thoroughly scientifically validated.

On the whole, **suggested interventions did not go beyond the scope of the list of ‘Systems of Care’ derived principles**. The key points re-iterated above span the whole spectrum of that list. Only the third item (‘providing culturally competent care and reducing unmet need and disparities in access to services’) was not picked up at all in any of the proposals.

## Discussion and conclusion

In this paper we’ve made a diagnostic analysis of the Belgian CAMHS system. The results of in-depth interviews with 10 high profile stakeholders were confirmed and complemented by roundtable discussions gathering input from 30 stakeholders. This study has been a realization of one of the essential steps in the development of a national mental health policy for children and adolescents as described by the WHO (i.e. undertake consultation and negotiation).

The results of this consultation process demonstrate that the problems besetting the Belgian CAMHS system go beyond highly visible dysfunctionalities, such as waiting lists and lack of crisis capacity. It clearly shows that the whole CAMHS system is under pressure and struggles with a cluster of interdependent problems. Demand has been on the increase, for which the care system is not able to cater, the core issue being the extreme fragmentation and compartmentalization resulting from powerful forces such as legal frameworks and vested interests. Interaction is hindered between organizations, sectors, professions and governmental levels, resources are scattered, and there is no overarching vision on care for people in this age group. The very long waiting lists are just one of the more conspicuous indicators of these burdens and inefficiencies.

Over the last decade several initiatives have been taken to deal with these pressures. However, services are constantly firefighting rather than taking a pro-active approach to implementing a well-designed and rational system of services and supports. Therefore, these innovations have not been able to bolster the adaptive capacity of the system as a whole. Past failures have also resulted in distrust between actors and sectors. The diagnostic analysis illustrates that a quick fix cannot be expected. The process of change is likely to be a lengthy one. The problem of a lack of child and mental health policy and problems such as fragmentation, poor coordination, a split between social and medical care and lack of a clear vision and evaluation framework has been described before in the international literature [2,6,11,14].

Identifying the problems is only a first step. It creates a sense of urgency that should be used to initiate a system reform of the Belgian CAMHS system. A dominant approach in the peer reviewed literature describes reforms on a systemic level, namely the “Systems of Care approach”. It is a whole systems approach that envisages a coherent process of reform at the practice level, the local level and the state level to deal with the kind of interconnected problems pointed out in the diagnostic analysis. The ‘Systems of Care Approach’ can be defined as *“a broad flexible array of effective services and supports for a defined multi-system involved population, which is organized into a coordinated network, integrates care planning and care management across multiple levels, is culturally and linguistically competent, builds meaningful partnerships with families and with youth at service delivery, management and policy levels, has supportive management and policy infrastructure, and is data-driven”*. In the US, large-scale studies were set up in the ‘90s, the Fort-Bragg and the Stark County studies [15,16] which aimed to evaluate the Systems of Care model. Changes were implemented such as different types of funding, a broader range of available services with both residential and outpatient care, and the provision of care coordination; this was compared with a control group. Positive effects were recorded in the experimental group, such as better access to care, better care coordination, and a higher level of patient satisfaction. However, the clinical and functional outcome parameters did not improve [8].

The ‘Systems of Care approach’ could help to build a framework for systemic reform in Belgium, adapted to the local context [11]. After all, the ten systemic interventions based on the “Systems of Care approach” that were proposed during the roundtable discussions appear to receive support from the Belgian stakeholders. In addition, there appeared to be considerable overlap between the suggestions offered by the interviewees and the proposals for interventions emerging from the roundtable discussions. The call for a sectorally and cross-sectorally more integrated system and for a broadening of the service offer (notably in the direction of more home and community-based services) were distinctly heard in both cases. Also other models such as CAPA (Choice and partnership approach) [17] should receive attention when designing a reform [8]. This is an innovative system developed by York and Kingsbury in England and currently implemented by CAMHS-teams in countries such as New Zealand, Canada and Belgium [17]. It aims to improve efficacy of CAMHS planning and to reduce waiting lists, while at the same time respecting the choice of the patients and families and engaging them in change [17].

Many countries are experimenting with new forms of care delivery at the local care organization level or the service delivery level, mainly under the form of community based intensive care models (e.g. intensive case management; wraparound; therapeutic foster care; multi-system therapy). Using these examples to nurture reforms in Belgium should be done with caution. After all, for each of these models, predominantly USA-based studies often show positive results for one or more of the measured parameters but the validity of these results is limited due to numerous methodological problems. Systematic reviews of good quality that studied these forms of care therefore concluded that no conclusions can be drawn, or, at best, that the results are promising but need to be supplemented with additional research [18-20].

Despite the merit of this study to highlight the systemic problems that should be addressed in future reform efforts it has several limitations mainly related to the study sample. A first limitation is the absence of young people with mental health problems in the study sample.

A second limitation is the absence of professionals from the adult mental health sector, although, in principle, they might have contributed with valuable insights (i.e. sharing experiences from reforms in adult mental health care; understanding the difficulties of young people transitioning into the adult system).

A third limitation is the relatively high number of child and adolescent psychiatrists. This was partly a result of the selection process, which was aimed at involving a sufficient number of child psychiatrists in view of their expertise, authority and the pivotal role they play in the current system. On the other hand, it has to be said that a high number of child psychiatrists accepted our invitation to the workshops, in which they played a very active role. Thus, a certain bias towards the perspective of child psychiatrists must be taken into account.

A fourth limitation is the relative small study sample. Given time and budgetary constraints we have limited the in-depth interviews to ten and the roundtable discussions to 15–20 each. It is unsure if this number was sufficient to capture the entire view of stakeholders on the organization of CAMHS in Belgium. Yet by using a balanced ‘purpose sampling technique’ we have tried to obtain information of people who are likely to provide the most relevant information in function of the research questions [21].

A fifth limitation is the qualitative nature of this study. As a consequence the synthetic headings and text fragments reflect stakeholder perceptions only, not verified facts. Nevertheless several strategies were used to increase the trustworthiness of the results (i.e. all conversations were audio-taped and transcribed; a theoretical framework was used to analyze results; the methods were triangulated: roundtable and in-depth interviews; the analysis was challenged by a second and third researcher) [22].

Despite these limitations this study offers valuable insights in the CAMHS system that could guide future reforms. It is recommended that proposed reforms are validated by a larger spectrum of stakeholders to ensure that the proposed recommendations are a response to the problems identified in the current research.

## Abbreviations

CAMHS: Child and adolescent mental health services; CAPA: Choice and partnership approach; KCE: Belgian Healthcare Knowledge Centre; WHO: World Health Organisation.

## Competing interests

The authors declare that they have no competing interests.

## Authors’ contributions

PV, RD, KB carried out the interviews. PV, RD, KB, ME, KV carried out the roundtables, the analysis of the interviews and roundtable discussions, participated in the design of the study and drafted the manuscript. All authors read and approved the final manuscript.
